# Socioeconomic and demographic predictors of extracurricular achievements among UK medical students (FAST study)

**DOI:** 10.1136/bmjopen-2025-103062

**Published:** 2025-08-08

**Authors:** Tomas Ferreira, Alexander M Collins, Arthur Handscomb, Benjamin French, Emily Bolton, Amelia Fortescue, Ella Plumb, Oliver Feng, The FAST Collaborative, Megan Fallows

**Affiliations:** 1 University of Bristol, Bristol, UK; 2 University of Bristol Medical School, Bristol, UK; 3 Queen Elizabeth Hospital, Woolwich, Lewisham and Greenwich NHS Trust, London, UK; 4 St George’s University Hospitals NHS Foundation Trust, London, UK; 5 Department of Mathematical Sciences, University of Bath, Bath, UK

**Keywords:** Education, Medical, MEDICAL EDUCATION & TRAINING, Health Education

## Abstract

**Abstract:**

**Objective:**

To investigate the relationship between demographic characteristics and extracurricular achievements among UK medical students.

**Design:**

National, cross-sectional survey.

**Setting:**

All 44 UK medical schools recognised by the General Medical Council.

**Participants:**

8,395 medical students.

**Outcomes:**

Binary indicators of extracurricular engagement, including PubMed-indexed authorship, academic presentations, quality improvement projects, leadership roles and academic prizes. Logistic regression models were used to explore associations with demographic and extracurricular achievement predictors.

**Results:**

Logistic regression analysis showed that students from private schools (OR 1.35, CI 1.20 to 1.53, p<0.0001) and those with a parent or sibling in medicine (OR 1.38, CI 1.12 to 1.69, p=0.002) had notably higher odds of participation in research. Ethnic disparities in raw extracurricular attainment were evident, but largely disappeared when adjusting for other predictors. Males were more likely to hold leadership roles and deliver oral presentations, but no gender differences were seen in publication rates.

**Conclusions:**

Significant disparities in extracurricular achievement exist among UK medical students, principally associated with gender, private schooling and familial links to medicine. Apparent ethnic differences were largely attenuated after adjustment for other variables, indicating socioeconomic factors as stronger predictors of engagement. Given the role of these achievements in postgraduate selection, targeted interventions by medical schools and professional bodies to widen access to funding, mentorship and structured guidance for *all* students, regardless of perceived advantage, may support equitable opportunity without undermining merit-based standards.

**STRENGTHS AND LIMITATIONS OF THIS STUDY**

- ⁠Factors Affecting Specialty Training examines the complex interplay of demographic factors that contribute to students’ extracurricular success, which is especially topical given recent research into the ‘awarding gap’, and growing levels of competition to entering specialist training in the UK.
- ⁠This study’s power benefitted from the presence of students from all 44 medical schools with actively enrolled students, constituting the second largest-ever sample of medical students in a UK-based study.
- The use of single-variable and multivariable logistic regression enabled us to identify which demographic factors were most strongly associated with the presence of extracurricular achievements among medical students.
- The cross-sectional nature of this investigation limits our ability to comment on how our findings might change, or have changed, over time.
- Recall bias, social desirability bias or both may have affected the responses obtained in this study.

## Introduction

 In the UK, the pathway to becoming a specialist consultant or general practitioner, the conventional endpoint of doctors’ careers, typically begins with the completion of the UK Foundation Programme (FP). The FP is a 2-year period during which newly qualified doctors rotate through various medical specialties within the National Health Service (NHS).^1 2^ Following the FP, doctors vie for entry into training programmes, a process that has become increasingly competitive in recent years due to rising numbers of applicants and limited availability of training positions.^3 4^ Consequently, distinguishing oneself through achievements outside the core medical school curriculum has become an essential component of successful applications to specialty training, commonly known overseas as ‘residency’.^5^ These ‘extracurricular’ activities—activities undertaken voluntarily beyond the standard medical school syllabus—include research authorship, academic presentations and leadership roles. Unlike core assessments, which are compulsory for all students, engagement in these activities typically requires additional initiative, mentorship and time commitment.

Despite the structured nature of medical training in the UK, disparities in academic and extracurricular achievements among medical students have emerged as significant issues. A recently acknowledged phenomenon, the ‘awarding gap’, refers to enduring disparities in academic performance between medical students from minority ethnic backgrounds compared with those from white backgrounds.^6^ This gap persists beyond undergraduate education into postgraduate assessments required for career progression.^7^ Less attention has been paid to how such disparities might manifest in medical students’ extracurricular achievements, which are increasingly responsible for success in securing a specialty training post.

There is evidence that disparities exist in UK medical students’ research involvement across different demographic groups, such as gender, ethnicity and socioeconomic status.^8^ Academic performance is also related to their preuniversity schooling, with state-educated students reportedly outperforming their peers who attended independent schools.^9^ Additionally, in the USA, researchers have highlighted an imbalance in the distribution of awards and accession to prestigious societies between medical students of different ethnic backgrounds.^10 11^ However, the evidence base remains incomplete, particularly concerning the complex interplay between multiple factors potentially influencing extracurricular success, including socioeconomic background, type of schooling and familial connections to the medical profession. Understanding these trends is critical as disparities in extracurricular engagement may translate into differences in competitiveness for specialty training applications and, ultimately, career progression.

The Factors Affecting Specialty Training (FAST) preference study’s novelty lies in its detailed examination of this underexplored area: the intersectionality of demographic influences on extracurricular achievements. The importance of fostering a culture of support cannot be overstated, especially given documented dissatisfaction surrounding the profession among UK students, with more than a third expressing an interest in emigrating or leaving the profession altogether.^12^ In this area, there appears to be a paucity of UK-based studies of adequate power which comprehensively address the relationship between a wide range of demographic factors and medical students’ extracurricular engagement and achievement.

In a companion analysis of the FAST study data, we present medical students’ specialty preferences and the factors underpinning this decision.^13^ In this present analysis, we aim to address the following research questions: How do demographic factors such as gender, ethnicity, type of schooling and family background influence extracurricular achievements among UK medical students? What are the implications of these disparities for specialty training applications and the broader medical profession? In addressing these questions, we seek to contribute to ongoing discussions about equity in medical education, and to inform strategies for supporting underrepresented students in their pursuit of successful medical careers.

## Methods

### Study design

The FAST study was a nationwide, multicentre, cross-sectional study investigating UK medical students. It followed a similar methodological framework to the Ascertaining the Career Intentions of UK Medical Students study,^14^ adhering closely to its published protocol.^15^ Students’ responses were recorded via an online survey platform. The 17-question survey employed a mix of Likert scale matrices, multiple-choice questions and free-text fields to capture a nuanced range of sentiments and improve data accuracy. Data collection began on 4 December 2023, ending on 1 March 2024.

The survey was divided into three sections. Section 1 collected background and demographic details (including confirmation of their student status), enquired about participants’ extracurricular achievements (according to published selection criteria for specialty training programmes) and obtained informed consent. Section 2 gathered information regarding respondents’ preferred specialty, their degree of certainty in this specialty preference and the extent of knowledge and guidance received in relation to that choice. Section 3 invited participants to evaluate the importance of various factors influencing their specialty decisions, with an additional option for free-text entries to capture other relevant factors. The complete questionnaire can be found in online supplemental material 1.

The survey was hosted on the General Data Protection Regulation (GDPR) compliant Qualtrics platform (Provo, Utah, USA), which supports both mobile and desktop devices. Participants’ responses were anonymised, stored securely on the Qualtrics server and subsequently extracted into a password-protected Microsoft Excel (Microsoft, California, USA) file, to which only the central study team had access.

### Participant recruitment and eligibility

Multiple streams of dissemination were employed to maximise survey responses, chiefly university mailing lists, student society social media platforms, conferences and personal and institutional social media profiles (such as Facebook, LinkedIn, Instagram and X). Invaluable to the national distribution of the survey was a network of approximately 200 FAST Collaborative members, representing all UK medical schools. Their role, for which all students undertaking a UK medical degree were eligible to apply, entailed liaising with their medical schools’ deans to gain approval to share the survey with their peers via official channels, as well as promoting the survey at regular intervals.

Eligibility to complete the survey was restricted only to those who were actively enrolled medical students at a UK medical school recognised by the General Medical Council (GMC) and Medical Schools Council. A list of eligible institutions and approved programmes is provided in online supplemental material 2. Newly GMC-approved medical schools whose programmes had yet to enrol their first cohort of students at the time of data collection were excluded from the study. Responses were received from all 44 eligible UK medical schools.

### Data processing and storage

To minimise the risk of duplicate entries, responses were each tied to a unique institutional email address. Duplicate email entries were removed before data analysis. In cases where duplicate entries contained varying responses, the latest entry was retained. Incomplete responses and those that lacked a valid institutional email address were excluded from the analysis to maintain data integrity.

### Outcome variables

Binary indicator variables were created for participation in the following activities: oral presentations, poster presentations, audit or quality improvement projects (QIPs), PubMed-indexed authorship (excluding collaborative authorship), leadership roles (regional or national), academic prizes (at medical school or national level) and holding an intercalated or previous degree. These were analysed individually and in aggregate to assess overall extracurricular engagement. Participation in these activities enhances individuals’ portfolios for increased chances of securing competitive specialty training posts.

### Data analysis

Data preprocessing and descriptive analyses were conducted using Microsoft Excel V.16.71 (Arlington, Virginia, USA). Graphs and tables were generated using GraphPad Prism V.10.3.0 (San Diego, California, USA), with percentages rounded to one decimal place. Statistical analyses were performed in RStudio V.1.4.1564 (Boston, Massachusetts, USA) using R V4.4.1 (Vienna, Austria).

For each extracurricular activity, multivariable logistic regression was used to assess associations with demographic and educational predictors. The *glm* function was used to calculate ORs, 95% CIs and p values. ORs were reported to two decimal places; p values<0.01 were rounded to two significant figures or denoted as p<0.0001. While individual p values were not adjusted, a Bonferroni-corrected threshold of 0.002 (0.05/25) was adopted for significance. All predictor variables were included simultaneously in each model using appropriate baseline categories to minimise the risk of confounding bias and strengthen the internal validity of our findings.

Study findings were reported in line with the Strengthening the Reporting of Observational Studies in Epidemiology guidelines.^16^

### Related analyses

In a separate FAST study publication, we explore medical students’ specialty preferences, perceived confidence in securing training posts and the factors shaping those decisions.^13^

### Patient and public involvement

The survey was developed following a review of previous literature and questionnaires exploring medical students’ and doctors’ perspectives on career planning. Feedback from senior clinical academics was incorporated to ensure that questions were clear, non-directive and sufficiently concise to maximise response rate without compromising data depth.

## Results

A total of 8395 students from all 44 UK medical schools participated in the survey (online supplemental material 4). The average number of responses per medical school was 191, with a median of 171 (IQR 124–224). A detailed breakdown of responses by medical school is available in the Supplemental Materials. The median participant age was 22 years (IQR 20–23). There was a notably lower response rate from students in the ‘Year 4 (not penultimate year)’ category compared with other year groups. This finding, shared by other similar investigations,^12^ is likely attributable to the smaller cohort of students undertaking intercalated degrees or 6-year programmes compared with those following traditional 5-year curricula. Among the respondents, 68.2% identified as female (n=5,727), 30.2% as male (n=2,537), 0.9% as non-binary (n=78) and 53 individuals preferred not to disclose their gender (table 1).

**Table 1:** Demographic characteristics of participants

Characteristic	Number	%
Ethnicity		
Asian or Asian British	2,532	30.2
Black, black British, Caribbean or African	491	5.9
Mixed or multiple ethnic groups	447	5.3
White	4,474	53.3
Other	354	4.2
Prefer not to say	97	1.2
Gender		
Female	5,727	68.2
Male	2,537	30.2
Non-binary	78	0.9
Prefer not to say	53	0.6
Level of education		
Postgraduate	1,505	17.9
Undergraduate	6,890	82.1
Previous schooling		
Comprehensive state school	4,061	48.4
Selective state school or grammar school	1,883	22.4
Private school (fee-paying)	2,180	26.0
Prefer not to say	271	3.2
Parent or sibling in medicine		
Yes	1,717	20.5
No	6,678	80.0
Fee status		
Home	7,305	87.0
EU/EEA	293	3.5
International (non-EU)	797	9.5
Year of study		
Year 1	1,258	15.0
Year 2	1,613	19.2
Year 3 (but not penultimate year)	1,688	20.1
Year 4 (but not penultimate or final year)	756	9.0
Penultimate year	1,759	21.0
Final year	1,321	15.7
Age		
Median (range)	22 (17–51)	

EU/EEA, European Union/European Economic Area.

### Full data set and missing values

Of the 8,395 students who participated, complete demographic data were available for 8035 participants. Students who selected ‘Prefer not to say’ for one or more key demographic items (ethnicity, gender or schooling) were excluded from regression analysis (n=360, 4.3%). A detailed breakdown of responses by medical school is available in online supplemental material 4.

### Extracurricular achievements

Participants were asked about their involvement in a variety of extracurricular engagements which, at the time of writing, confer additional points when applying for specialty training programmes.^17^ Students were able to select multiple activities, and each achievement was recorded as a separate binary variable. These include research, quality improvement, presentations, leadership and academic prizes. Students were able to select all activities that applied to them. While these criteria were previously central to the application process for the Academic (or Specialised) Foundation Programme (AFP/SFP), recruitment to this scheme has since shifted to a preference-informed rather than merit-based model.^18^

With regard to research engagement, 6.5% of students (n=547) had coauthored a PubMed-indexed publication, 3.7% (n=303) had been first author and 3.9% (n=323) were cited collaborative authors. Involvement in audit or QIPs was reported by 17.9% of participants, while 20.5% had delivered a poster presentation and 15.8% had given an oral presentation.

In terms of leadership, 12.7% had held a local or regional leadership role and 3.4% a national position. Academic recognition was also reported, with 19.7% (n=1613) of students having received a merit or prize in medical school examinations, and 4.2% (n=350) holding a national or international award (eg, conference or essay prizes). Additionally, approximately one-third of respondents (33.5%, n=2,809) had completed either an intercalated or previous degree.

**Figure 1:**
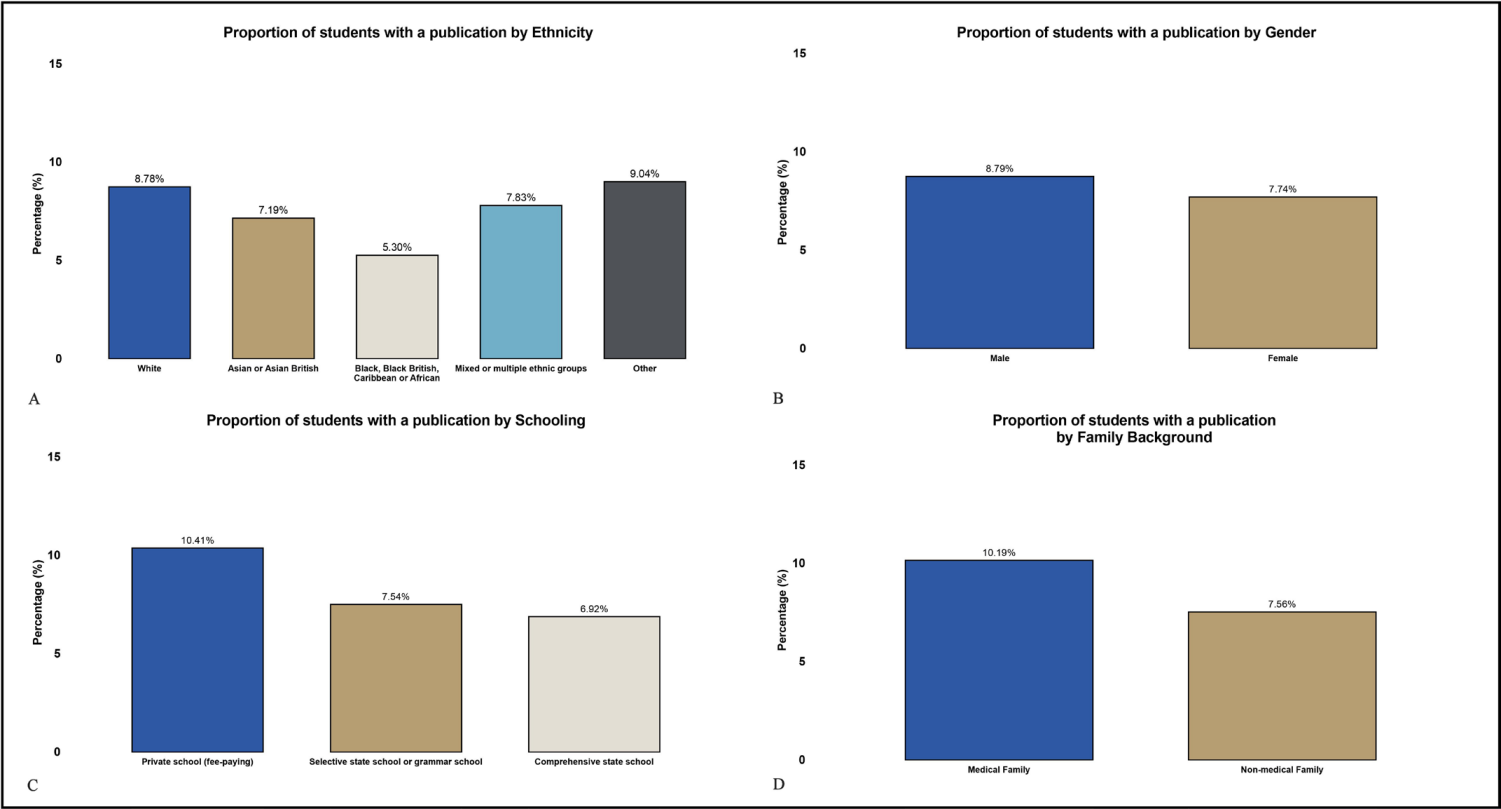
Proportion of medical students with a publication by ethnicity, gender, schooling and family background. This figure displays the proportion of medical students who have authored a publication, segmented by key demographic factors. (A) shows differences by ethnicity. (B) compares schooling backgrounds. (C) presents publication rates by gender. (D) compares students with and without a doctor in the family.

A summary of participation rates across all categories is shown in table 2 and figure 1. Percentages in table 2 reflect the proportion of respondents reporting participation in each activity. As multiple selections were permitted, totals exceed 100%.

**Table 2:** Distribution of medical students by participation in extracurricular activities (%)

Extracurricular activities	%
First author on a PubMed-indexed publication	3.7
Coauthor on a PubMed-indexed publication (non-first author)	6.5
Cited collaborative author on a PubMed-indexed publication	3.9
Involvement in an audit/quality improvement project	17.9
Delivered a poster presentation	20.5
Delivered an oral presentation	15.8
Recipient of a national or international prize related to medicine (eg, essay or conference prizes)	4.2
Held a national leadership role	3.4
Held a regional or local leadership role	12.7
Recipient of a medical school examination merit/prize(s)	19.3
None of the above	51.1

### Multivariable logistic regression analysis of extracurricular achievements

When controlling for all other demographic and educational predictors, males were significantly more likely than females to participate in extracurricular activities (OR 1.16, CI 1.05 to 1.29, p=0.005) including oral presentations (OR 1.25, CI 1.1 to 1.43, p=0.0007), leadership roles (OR 1.2, CI 1.04 to 1.38, p=0.001) and audits/QIPs (OR 1.2, CI 1.05 to 1.37, p=0.008). No gender differences were seen in publication rates (OR 1.08, CI 0.9 to 1.3, p=0.39).

A higher proportion of white students (50.9%) reported extracurricular achievements than those from Asian (46.9%) or black (41.6%) backgrounds. However, in fully adjusted logistic regression models, these differences were attenuated. Compared with white students, ORs for overall extracurricular attainment were 1.05 (95% CI 0.94 to 1.19, p=0.34) for Asian students and 1.27 (95% CI 1.02 to 1.56, p=0.03) for black students. Inclusion of ethnicity did not significantly improve overall model fit (χ²=5.97, df=4, p=0.2), suggesting that disparities by ethnicity may be largely mediated by other demographic factors. White students remained significantly more likely to receive academic prizes than Asian (OR 1.27, 95% CI 1.10 to 1.45, p=0.001) and black (OR 1.59, 95% CI 1.20 to 2.08], p=0.001) peers. Asian students were more likely to have held leadership positions (OR 1.28, 95% CI 1.09 to 1.49, p=0.002).

In every activity evaluated, levels of participation and attainment were found to be significantly higher among students educated at private (fee-paying) schools (OR 1.35, CI 1.2 to 1.53, p<0.0001) and grammar schools (OR 1.18, CI 1.04 to 1.33, p=0.008) than those who attended comprehensive state schools, controlling for all other characteristics. Particularly striking were the disparities in involvement in research authorship (OR 1.41, CI 1.14 to 1.74, p=0.001) and leadership experiences (OR 1.5, CI 1.27 to 1.76, p<0.0001). Privately educated students were also found to have a stronger track record in audits/QIPs (OR 1.23, CI 1.08 to 1.39, p<0.002). They also reported higher rates of medical school examination merits or prizes (OR 1.28, CI 1.12 to 1.48, p=0.0004) (online supplemental figure 5).

**Figure 2:**
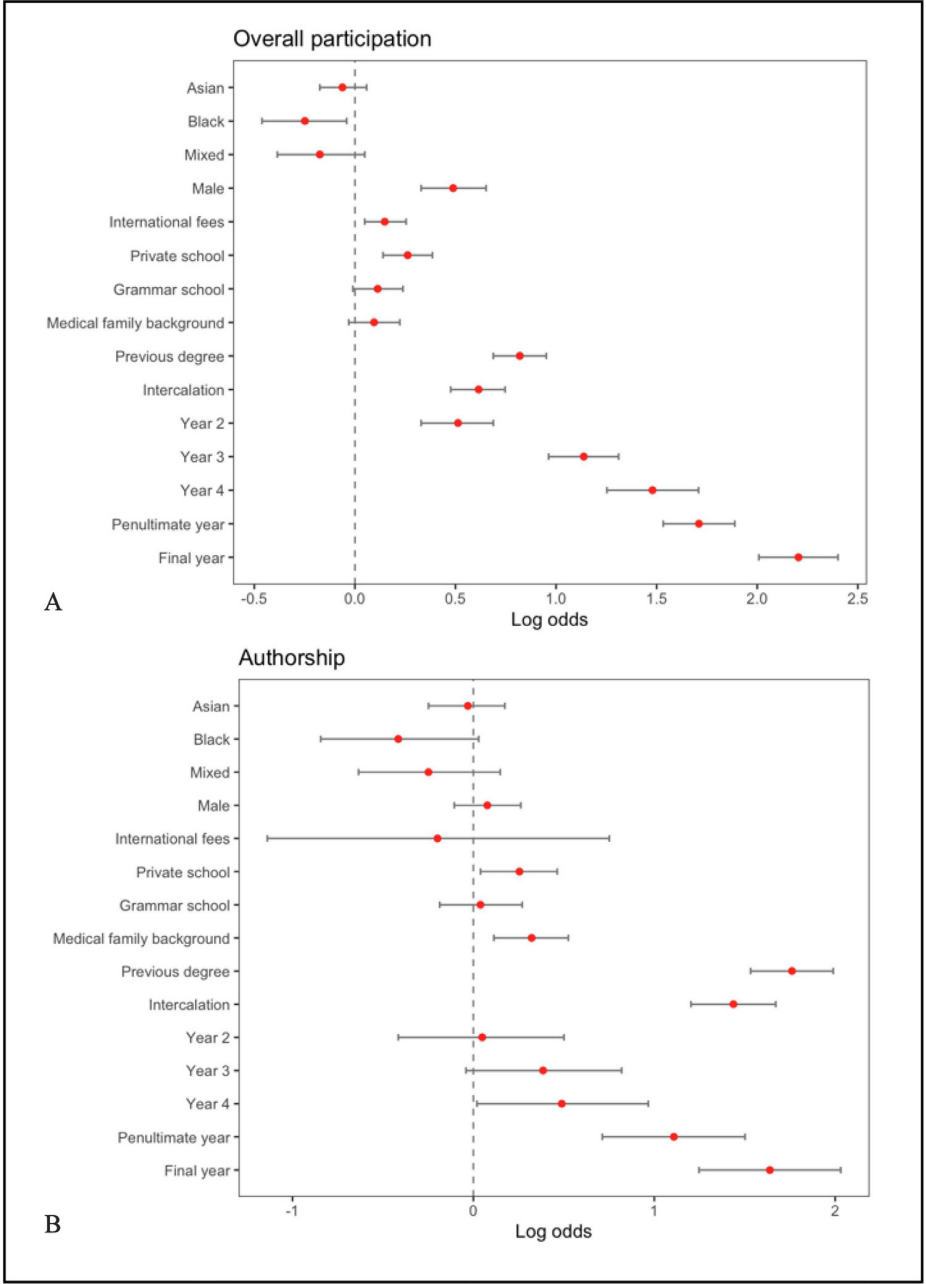
Adjusted ORs for predictors of extracurricular and research participation among UK medical students. Multivariable logistic regression models evaluating associations between key predictors and (A) overall extracurricular engagement and (B) authorship of PubMed-indexed research. Models adjusted for gender (baseline: female), ethnicity (baseline: white), schooling background (baseline: comprehensive state school), intercalation status, previous degree, year of study (baseline: Year 1) and family in medicine (baseline: no parent/sibling in medicine). ORs >1 indicate increased likelihood of participation relative to the reference category. Red points represent point estimates; horizontal bars indicate 95% CIs.

Moreover, our analysis extended further to consider the influence of having a parent or sibling in medicine. This familial factor was associated with higher participation and attainment research authorship (OR 1.38, CI 1.12 to 1.69, p=0.002) but overall levels of extracurricular engagement were not significantly higher among such students (OR 1.10, CI 0.97 to 1.25, p=0.12) (online supplemental figure 5). Excluding students with intercalated or pre-medicine degrees further emphasised the effect that a medic in one’s family has on the likelihood of coauthoring publications (OR 1.41, CI 1.10 to 1.80, p=0.006) (figure 2, table 3).

**Table 3:** Adjusted ORs for demographic and educational predictors of overall extracurricular participation (left) and PubMed-indexed authorship (right) among UK medical students

	Overall extracurricular participation				Authorship			
	OR	Lower	Upper	P value	OR	Lower	Upper	P value
Ethnicity								
White	–	–	–	–	–	–	–	–
Asian	0.94	0.84	1.06	0.31	0.97	0.78	1.19	0.76
Black	0.78	0.63	0.96	0.02	0.66	0.43	1.03	0.066
Mixed	0.84	0.68	1.05	0.12	0.78	0.53	1.16	0.22
Other	1.01	0.78	1.3	0.95	1.11	0.73	1.7	0.61
Fee status								
UK	–	–	–	–	–	–	–	–
Non-UK	1.63	1.39	1.92	**<0.0001**	1.77	1.38	2.26	**<0.0001**
Gender								
Female	–	–	–	–	–	–	–	–
Male	1.16	1.05	1.29	0.0047	1.08	0.9	1.3	0.39
Non-binary	1.5	0.9	2.49	0.12	0.82	0.32	2.12	0.68
Schooling								
Comprehensive state school								
Private fee-paying school	1.3	1.15	1.47	**<0.0001**	1.29	1.04	1.59	0.018
Grammar school	1.12	0.99	1.27	0.066	1.04	0.83	1.31	0.72
Medic in the family								
No	–	–	–	–	–	–	–	–
Yes	1.1	0.97	1.25	0.12	1.38	1.12	1.69	0.0022
Previous degree and intercalation								
None	–	–	–	–	–	–	–	–
Previous degree	2.27	1.99	2.59	**<0.0001**	5.82	4.63	7.31	**<0.0001**
Intercalated	1.85	1.61	2.11	**<0.0001**	4.21	3.33	5.32	**<0.0001**
Year of study								
Year 1	–	–	–	–	–	–	–	–
Year 2	1.67	1.39	1.99	**<0.0001**	1.05	0.66	1.65	0.84
Year 3	3.12	2.62	3.71	**<0.0001**	1.47	0.96	2.27	0.079
Year 4	4.39	3.5	5.52	**<0.0001**	1.63	1.02	2.63	0.043
Penultimate year	5.53	4.63	6.61	**<0.0001**	3.03	2.04	4.49	**<0.0001**
Final year	9.07	7.45	11.04	**<0.0001**	5.15	3.48	7.62	**<0.0001**

Multivariable logistic regression models were adjusted for gender (baseline: female), ethnicity (baseline: white), schooling background (baseline: comprehensive state school), intercalation status, previous degree, year of study (baseline: year 1) and medical family background (baseline: no parent/sibling in medicine). ORs >1 indicate increased likelihood of the outcome relative to the reference category. Statistically significant results (Bonferroni-corrected threshold p<0.002) are bolded.

## Discussion

The FAST study, with its large sample size, offers a detailed analysis of UK medical students’ extracurricular achievements and the demographic factors influencing these outcomes. Comprehensive evaluation of these data highlighted persistent disparities in extracurricular achievement, driven by gender, ethnicity, schooling and family background. These findings highlight the complex, multifactorial interplay that contributes to the extracurricular success of medical students, with broader implications for their future careers.

### Gender and ethnicity disparities

Our study identified significant gender inequalities, with male students more commonly holding leadership roles and delivering oral presentations, supporting existing literature on gender imbalances in academic medicine leadership.^19^ However, no significant differences were found in authorship rates between genders, suggesting progress towards more equitable distribution of research opportunities despite previously reported differences.^20^

Ethnic disparities were also pronounced, but discussion of these requires nuance. Our analysis showed white students generally achieving higher levels of extracurricular involvement than their peers from black or Asian backgrounds, most markedly in research authorship and the receipt of academic prizes. These findings echo those from other key studies on differential attainment within medical education, notably Brown *et al*, who highlighted significant differences in examination performance between ethnic groups at UK medical schools.^6^ Differential attainment in medical education may, therefore, be more far-reaching than previously documented.

However, while ethnicity constitutes an eminently important factor affecting students’ success, both at medical school and beyond,^21^ our analysis signals that differences in *extracurricular* attainment may be better explained by a combination of factors, including gender, year of study, type of schooling and family background, rather than ethnicity alone. Factors such as ethnicity often intersect with other demographic characteristics which collectively shape students’ experiences at medical school and their access to opportunities within medical education. For instance, students from private schools—who are disproportionately white—were found to have significantly higher levels of achievement across all domains investigated. It is therefore important to consider the role of institutional support and access to opportunities in driving these outcomes. This provides important context to the discussion around the ‘awarding gap’^6^ and could inform more effective, targeted interventions aimed at addressing the root causes of these disparities.

### Impact of schooling and family background

The influence of schooling and family background on extracurricular achievements is striking. Privately educated students, who are over-represented in medical school cohorts,^22^ consistently outperformed their peers from state schools, particularly in research authorship, leadership experience and academic prizes. This suggests that advantages conferred by private education, such as existing networks, resources and tailored support, extend well beyond the classroom and into the extracurricular domain, providing these students with an edge in their future career prospects. Still, it is important to note that school type is an imperfect proxy for socioeconomic status and may obscure the diversity of students’ financial circumstances or broader social advantage.

Students with a parent or sibling in medicine were also more likely to excel in research-related achievements. This *familial advantage* likely reflects greater access to guidance, mentorship and opportunities that relate directly to a medical career. Such dynamics, often regarded as nepotism, raise concerns about the perpetuation of privilege, or ‘cumulative advantage’, within the medical profession.^23^,^27^ Previous works, both from the UK and internationally, have highlighted the apparent role that this factor plays in admission to medical school, suggesting that this advantage may have implications at all career stages.^27 28^ However, differences in student motivation, personal aspirations and opportunistic access to extracurricular activities outside the university environment may also contribute to these outcomes.

Both this work and its companion publication^13^ corroborate previous findings detailing the impact of the ‘hidden curriculum’—the unspoken or informal lessons, values and perspectives that students absorb through their educational experiences.^29^ This has profound implications for medical education as it may be contributing to published differences in students’ career intentions between institutions.^30^

### Broader implications and future directions

When discussing these disparities in extracurricular success and seeking meaningful change thereto, it is important not to undermine the drive and determination of individual students who have excelled in these areas. It remains important to maintain elements of meritocracy in selection processes, so as not to dissuade medical students from being actively involved in endeavours valuable to their development as future doctors and academics. Systems abroad use merit-based systems emphasising both academic success and extracurricular criteria.^31 32^ In planning our future efforts to address the disparities highlighted in this study, we must avoid falling into the trap of reducing these efforts to mere ‘tick-box’ exercises and tokenism. Recent critiques surrounding the changes to FP and AFP recruitment processes stress the importance of maintaining meritocracy.^18 33 34^

⁠Interventions should focus on levelling the playing field for students from less privileged backgrounds, ensuring that all students, no matter their socioeconomic status or family background, have equal access to opportunities that influence specialty training success. One key area for improvement is the level of financial support for medical students, particularly for those from lower socioeconomic backgrounds. Increasing access to funding for subsistence, ideally in the form of bursaries and stipends, would allow these students to better engage with their coursework and extracurricular pursuits, reducing reliance on part-time employment to support themselves.^35^ This approach, for which the UK’s medical union is currently advocating, promotes true equity, contrasting the removal of accolades from extracurricular achievements, which risks fostering mediocrity rather than excellence.^36^

A key factor limiting extracurricular engagement, particularly for students from lower socioeconomic backgrounds, is financial constraint. The financial burden of undertaking a medical degree has ballooned in recent years, amid rising living costs and a lack of targeted funding for research, conferences and leadership initiatives, disproportionately disadvantages students who must work alongside their studies. This financial burden restricts engagement in unpaid extracurricular activities, contributing to disparities in research output, leadership roles and academic recognition. Prior studies have demonstrated that medical students from disadvantaged backgrounds are more likely to work part-time, limiting their ability to participate in academic and professional development opportunities.^35 37^

While recent efforts have sought to address inequities in training selection, such as the removal of academic scoring from UK FP and, more recently, specialised FP recruitment, such measures do not resolve structural barriers and may, in fact, exacerbate disparities. Eliminating academic components from selection criteria risks disincentivising engagement in research and leadership while lowering overall standards for clinical academia. Recent critiques have argued that removing academic selection from foundation training recruitment represents a fundamental step backwards, undermining meritocratic selection without addressing the root causes of inequity.^33^ Instead, a more effective solution would be to expand financial support for all students, ensuring equitable access to the very activities that are rewarded in specialty training selection. Increases in funding for subsistence should therefore be coupled with bursaries for conference attendance, funded research placements and structured mentorship schemes which would allow *all* students to compete on merit rather than financial privilege. With funding secured, there would be an even greater onus on meritocratic medical selection—ensuring that training places are awarded based on demonstrated ability, rather than removing meaningful selection criteria under the guise of fairness. Medical schools and professional bodies must focus on levelling the playing field by increasing opportunity, rather than lowering the bar.

In many countries, specialty training is preceded by only 1 year of work, typically termed an internship. The UK’s adoption of a 2-year FP is relatively unusual in this regard and may partly explain why UK medical students often defer career-related decisions until after graduation.^38^ However, in the context of intensifying competition for specialist training places,^3 4^ there is clear value in supporting students to make these decisions earlier. While many UK medical schools offer some form of careers education and informal mentoring, these efforts are often inconsistent, inadequately resourced and lack meaningful long-term follow-up.^30 39^ Strengthening such initiatives through the establishment of formal, well-supported mentorship programmes connecting students with experienced clinicians for sustained career guidance, leadership development and research exposure could significantly bolster their impact. As shown in other settings,^40^ embedding structured networking with established professionals within these programmes may better support students, particularly those without access to informal guidance through personal connections.

Similarly, structured undergraduate research programmes could play a key role. Schemes that offer targeted teaching and academic supervision provide students with meaningful exposure to research, resulting in tangible outputs such as coauthored publications and transferable skills aligned with specialty portfolio criteria. These schemes have demonstrated high levels of student satisfaction and perceived skill development.^41^ While students from ‘medical families’ may already benefit from informal networks and guidance, providing similar structured support to those without familial advantages could help bridge the gap in extracurricular achievements and career confidence.⁠ While students from medical families may already benefit from informal access to such networks and opportunities, formalised support structures—open to *all* students—could help level the playing field for those without these advantages. This responsibility extends beyond medical schools: royal colleges and professional associations are well placed to coordinate and resource such initiatives, ensuring a more equitable distribution of opportunity across the future workforce.

Additionally, introducing mandatory career guidance sessions and specialty exposure opportunities from early in medical school could be invaluable. Initiatives should include rotations, shadowing opportunities and workshops designed to convey students a realistic view of different specialties from their first year. Providing early and comprehensive exposure to various medical fields will empower students to make more informed career-related decisions, reducing uncertainty and increasing confidence in their chosen paths. This proactive approach, which would complement mentorship schemes, would better equip students to navigate the complexities of specialty training applications and ultimately contribute to a more equitable and fulfilled medical workforce.^39^

### Limitations

Although this study provides valuable insights into the extracurricular achievements and demographic influences among UK medical students, several limitations must be acknowledged. First, its cross-sectional design captures data at a single point in time, limiting our ability to draw causal inferences about relationships between demographic factors and extracurricular achievements. Longitudinal studies would be required to track changes in extracurricular engagement over time and better understand causal pathways. Second, the study relies on self-reported data, which may be subject to recall bias and social desirability bias. However, the anonymous nature of the survey likely mitigated some of these biases. It is also not possible to verify the accuracy of participants’ self-reported achievements. Third, while we adjusted for multiple demographic variables in our analysis, there may be other unmeasured factors that contribute to the disparities observed. For example, differences in medical schools’ resources and support systems could influence extracurricular involvement but were not specifically addressed in this study. As participation was voluntary and the survey was predominantly disseminated online, the response rate is unknown. Furthermore, the sample exhibited a gender imbalance, which may have influenced observed trends. International students were also overrepresented relative to UK medical school admission caps, possibly due to the inclusion of institutions that exclusively admit international students. Additionally, ethnic groupings in this study were broad and may not fully capture the heterogeneity within categories. While this approach aligns with prior research, it may overlook important subgroup differences.

Finally, the use of schooling as a proxy for students’ socioeconomic status was the most intrusive measure deemed feasible without deterring participation. A more precise indicator, such as household income or deprivation score, as per previous non-survey-based UK medical student-focused research,^6 23^ would have provided a better assessment of socioeconomic status. However, these measures were omitted due to concerns that they might reduce the response rate and compromise the study’s validity. Future research could consider alternative methods for capturing this important variable without impacting participation.

## Conclusion

This study describes significant disparities in extracurricular achievement among UK medical students, strongly influenced by gender, schooling and family background. While raw ethnic differences were evident, our adjusted analyses suggest that socioeconomic and structural factors may play a more central role in extracurricular success. These findings emphasise the need for structural reform, not by removing academic merit from selection processes, but by ensuring that all students have equitable access to the opportunities that underpin success. Efforts from medical schools and professional bodies should focus on expanding financial support, embedding structured mentorship schemes and delivering early career guidance across the board—avoiding tokenism and fostering genuine meritocracy. Future research should build on these findings through longitudinal and qualitative work to further address barriers to opportunity. By levelling access, not standards, we can cultivate a more equitable and capable future medical workforce.

## Supplementary material

10.1136/bmjopen-2025-103062 online supplemental file 1

10.1136/bmjopen-2025-103062 online supplemental file 2

10.1136/bmjopen-2025-103062 online supplemental file 3

10.1136/bmjopen-2025-103062 online supplemental file 4

10.1136/bmjopen-2025-103062 online supplemental file 5

## Data Availability

Data are available upon reasonable request. All data relevant to the study are included in the article or uploaded as supplementary information.
