# Supplementary material for: Socioeconomic and demographic predictors of extracurricular achievements among UK medical students (FAST study)
Source: BMJ Open. 2025 Aug 8;15(8):e103062. doi: 10.1136/bmjopen-2025-103062 (PMC12336483; doi:10.1136/bmjopen-2025-103062)
Supplement: online supplemental file 2 [file bmjopen-15-8-s002.pdf]

GMC Approved Medical Schools

Anglia Ruskin University School of Medicine
Aston University Medical School
Barts and The London School of Medicine and Dentistry
Brighton and Sussex Medical School
Brunel University London, Brunel Medical School
Cardiff University School of Medicine
Edge Hill University Medical School
Hull York Medical School
Imperial College London Faculty of Medicine
Keele University School of Medicine
Kent and Medway Medical School
King's College London GKT School of Medical Education
Lancaster University Medical School
Newcastle University School of Medical Education
Norwich Medical School
Plymouth University Peninsula Schools of Medicine and Dentistry
Queen's University Belfast School of Medicine
ScotGEM (A combination of St Andrew's and Dundee)
St George's, University of London
Swansea University Medical School
The University of Edinburgh Medical School
Ulster University, School of Medicine
University College London Medical School
University of Aberdeen School of Medicine and Dentistry
University of Birmingham College of Medical and Dental Sciences
University of Bristol Medical School
University of Buckingham Medical School
University of Cambridge School of Clinical Medicine
University of Central Lancashire School of Medicine
University of Dundee School of Medicine
University of Exeter Medical School
University of Glasgow School of Medicine
University of Leeds School of Medicine
University of Leicester Medical School
University of Liverpool School of Medicine
University of Manchester Medical School
University of Nottingham - Lincoln Medical School
University of Nottingham School of Medicine
University of Oxford Medical Sciences Division
University of Sheffield Medical School
University of Southampton School of Medicine
University of St Andrews School of Medicine

University of Sunderland School of Medicine
University of Warwick Medical School
