# Supplementary figures and images for: Socioeconomic and demographic predictors of extracurricular achievements among UK medical students (FAST study)
Source: BMJ Open. 2025 Aug 8;15(8):e103062. doi: 10.1136/bmjopen-2025-103062 (PMC12336483; doi:10.1136/bmjopen-2025-103062)

# Total responses by medical school

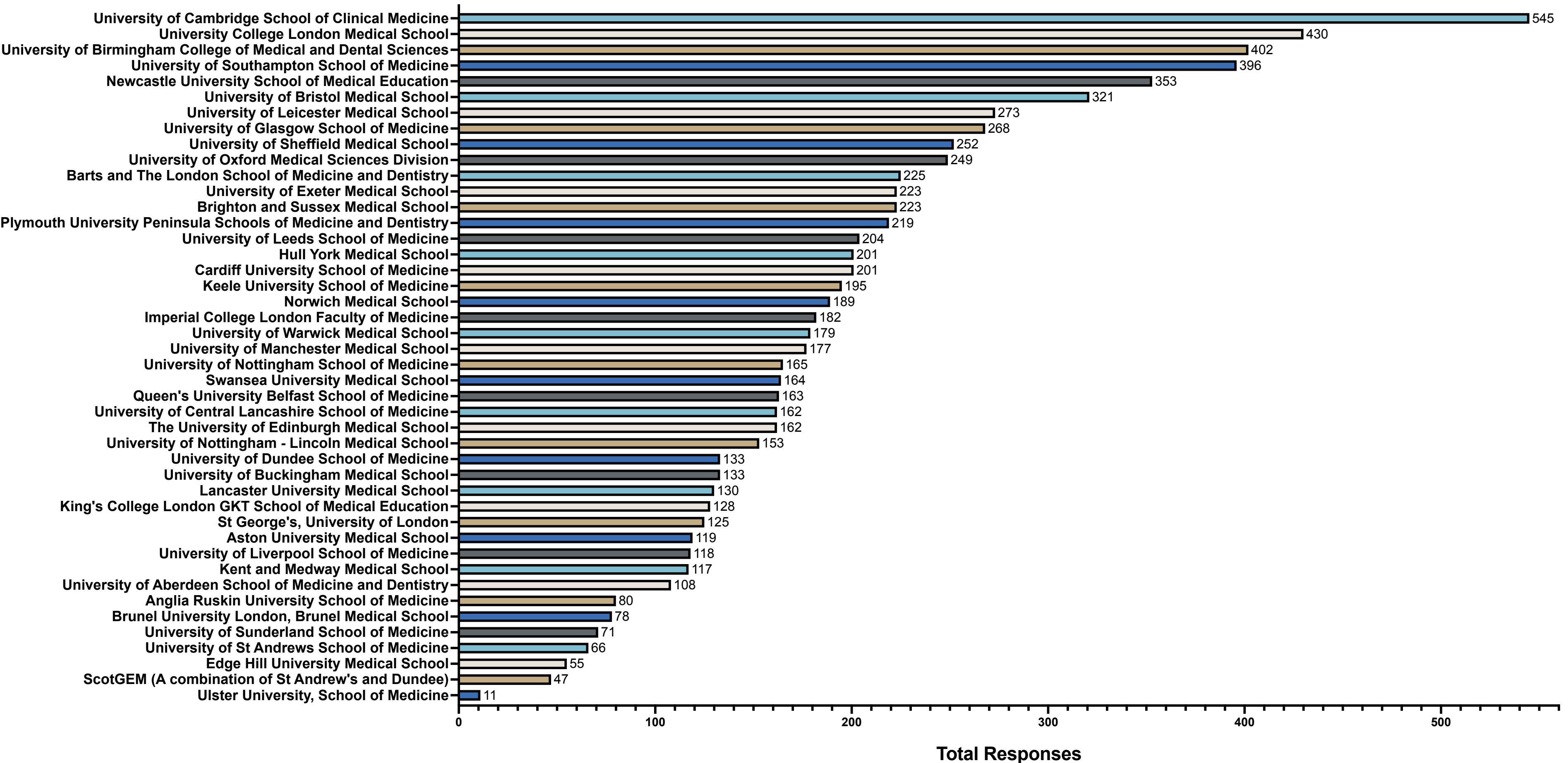

Supplement: online supplemental file 4 [file bmjopen-15-8-s004.pdf]
