# Supplementary material for: Socioeconomic and demographic predictors of extracurricular achievements among UK medical students (FAST study)
Source: BMJ Open. 2025 Aug 8;15(8):e103062. doi: 10.1136/bmjopen-2025-103062 (PMC12336483; doi:10.1136/bmjopen-2025-103062)
Supplement: online supplemental file 5 [file bmjopen-15-8-s005.docx]

| **Characteristic** | | **First author** | **Co-author** | **Cited Collaborative author** | **Involvement in an audit/QI project** | **Poster presentation** | **Oral presentation** | **Recipient of a national or international prize** | **National leadership role** | **Regional or local leadership role** | **Medical school examination merit/prize(s)** | **None of the above** |
| --- | --- | --- | --- | --- | --- | --- | --- | --- | --- | --- | --- | --- |
| *Ethnicity* | |  |  |  |  |  |  |  |  |  |  |  |
|  | Asian or Asian British | 3.36% | 5.81% | 4.38% | 16.35% | 20.18% | 14.10% | 4.42% | 4.38% | 14.73% | 16.19% | 53.28% |
|  | Black, Black British, Caribbean or African | 1.63% | 4.48% | 2.65% | 13.24% | 17.92% | 14.46% | 4.48% | 3.26% | 12.22% | 12.02% | 58.86% |
|  | Mixed or multiple ethnic groups | 4.03% | 6.49% | 4.70% | 19.69% | 18.79% | 13.87% | 4.25% | 3.58% | 13.65% | 19.91% | 51.23% |
|  | White | 4.00% | 6.95% | 3.53% | 19.11% | 20.90% | 17.08% | 3.91% | 2.82% | 11.35% | 22.17% | 49.02% |
|  | Other | 3.67% | 7.91% | 3.67% | 16.95% | 22.60% | 16.10% | 4.80% | 3.67% | 14.69% | 13.28% | 50.85% |
|  | Prefer not to say | 5.15% | 10.31% | 7.22% | 17.53% | 19.59% | 19.59% | 5.15% | 5.15% | 13.40% | 22.68% | 54.64% |
| *Gender* | |  |  |  |  |  |  |  |  |  |  |  |
|  | Female | 3.60% | 6.02% | 3.67% | 17.30% | 19.82% | 14.88% | 4.14% | 3.07% | 11.84% | 19.02% | 52.05% |
|  | Male | 3.78% | 7.45% | 3.98% | 19.08% | 21.92% | 17.93% | 4.10% | 4.18% | 14.39% | 19.79% | 49.23% |
|  | Non-binary | 3.85% | 5.13% | 6.41% | 16.67% | 19.23% | 17.95% | 7.69% | 5.13% | 23.08% | 21.79% | 43.59% |
|  | Prefer not to say | 5.66% | 16.98% | 13.21% | 20.75% | 20.75% | 16.98% | 5.66% | 1.89% | 11.32% | 20.75% | 54.72% |
| *Level of education* | |  |  |  |  |  |  |  |  |  |  |  |
|  | Postgraduate | 7.57% | 14.15% | 7.31% | 23.79% | 28.77% | 22.26% | 5.45% | 3.85% | 13.36% | 21.86% | 39.53% |
|  | Undergraduate | 2.82% | 4.85% | 3.09% | 16.56% | 18.64% | 14.44% | 3.89% | 3.32% | 12.57% | 18.72% | 53.67% |
| *Previous schooling* | |  |  |  |  |  |  |  |  |  |  |  |
|  | Comprehensive state school | 3.25% | 5.44% | 3.62% | 16.57% | 19.28% | 15.12% | 3.69% | 2.46% | 9.92% | 18.03% | 54.25% |
|  | Selective state school or grammar school | 3.51% | 6.21% | 3.77% | 18.27% | 19.65% | 15.51% | 4.14% | 3.61% | 14.39% | 18.85% | 51.57% |
|  | Private school (fee-paying) | 4.68% | 8.35% | 4.04% | 20.18% | 23.44% | 17.61% | 5.28% | 4.95% | 16.38% | 22.02% | 45.09% |
|  | Prefer not to say | 2.95% | 9.96% | 6.27% | 15.50% | 19.56% | 14.76% | 2.58% | 4.06% | 13.28% | 19.19% | 50.18% |
| *Parent or sibling in Medicine* | |  |  |  |  |  |  |  |  |  |  |  |
|  | Yes | 4.89% | 8.15% | 5.01% | 19.16% | 22.25% | 17.36% | 5.36% | 4.43% | 13.74% | 20.73% | 48.40% |
|  | No | 3.35% | 6.09% | 3.55% | 17.52% | 19.99% | 15.45% | 3.86% | 3.16% | 12.44% | 18.91% | 51.84% |
| *Fee status* | |  |  |  |  |  |  |  |  |  |  |  |
|  | Home | 3.35% | 5.93% | 3.20% | 17.25% | 19.53% | 15.09% | 3.93% | 2.90% | 11.27% | 19.26% | 52.47% |
|  | EU/EEA | 8.53% | 13.99% | 11.26% | 26.62% | 34.13% | 29.69% | 8.53% | 6.48% | 25.94% | 25.60% | 33.45% |
|  | International (non-EU) | 4.77% | 9.16% | 7.03% | 20.20% | 23.84% | 17.69% | 4.77% | 7.03% | 21.08% | 17.19% | 45.42% |
| *Year of study* | |  |  |  |  |  |  |  |  |  |  |  |
|  | Year 1 | 1.59% | 2.31% | 1.67% | 2.23% | 11.84% | 12.16% | 1.67% | 0.95% | 4.69% | 0.87% | 78.46% |
|  | Year 2 | 1.05% | 2.98% | 2.36% | 4.34% | 13.83% | 11.53% | 1.36% | 1.98% | 7.56% | 11.59% | 67.70% |
|  | Year 3 (but not penultimate year) | 1.78% | 3.61% | 2.49% | 10.96% | 15.52% | 11.91% | 2.31% | 2.55% | 12.68% | 21.27% | 54.09% |
|  | Year 4 (but not penultimate or final year) | 3.57% | 6.08% | 5.03% | 18.25% | 21.83% | 17.59% | 5.69% | 4.63% | 15.34% | 27.78% | 40.87% |
|  | Penultimate year | 5.00% | 8.93% | 5.40% | 28.99% | 25.64% | 17.40% | 5.86% | 4.78% | 17.06% | 24.28% | 37.24% |
|  | Final year | 9.54% | 15.59% | 6.74% | 43.00% | 35.35% | 26.57% | 9.24% | 6.13% | 19.38% | 32.17% | 25.51% |

**Supplemental Material 5** - Distribution of Extracurricular Participation Among UK Medical Students by Demographic Characteristics
