# Supplementary material for: Socioeconomic and demographic predictors of extracurricular achievements among UK medical students (FAST study)
Source: BMJ Open. 2025 Aug 8;15(8):e103062. doi: 10.1136/bmjopen-2025-103062 (PMC12336483; doi:10.1136/bmjopen-2025-103062)
Supplement: Supplementary file 6 [file bmjopen-2025-103062.reviewer_comments.pdf]

PEER REVIEW HISTORY

BMJ Open publishes all reviews undertaken for accepted manuscripts. Reviewers are asked to complete a checklist review form and are provided with free text boxes to elaborate on their assessment. These free text comments are reproduced below.

ARTICLE DETAILS

Title (Provisional)

Socioeconomic and demographic predictors of extracurricular achievements among UK medical students (FAST Study)

Authors

Ferreira, Tomas; Collins, Alexander M.; Feng, Oliver; French, Benjamin; Plumb, Ella; Handscomb, Arthur; Fortescue, Amelia; Bolton, Emily; ., the FAST Collaborative

---

VERSION 1 - REVIEW

**Reviewer**	**1**
**Name**	**Spain, Eimear**
**Affiliation**	**University of Limerick**
**Date**	**16-Jan-2025**
**COI**	**None**

A really well written article providing valuable insights into the socioeconomic and demographic predictors of extracurricular achievements, an important topic given their role in selection for specialist training programmes. This will be of interest to readers.

Include a sentence in the abstract outlining why extracurricular achievement is important, that is why it's important to look at socioeconomic and demographic influences.

While there is a recognition in the limitations section of the fact that attendance at private school is not necessarily indicative of socio economic standing, this should be acknowledged earlier in the discussion.

Overall, a really well written article making use of strong data.

**Reviewer**	**2**
**Name**	**Gishen, Faye**
**Affiliation**	**UCL**

Date **16-Jan-2025**

COI **FG is Director of a UK medical school and Head of an MBBS Programme.**

This is an important paper with relevance to the community of practice- undergraduate medical education. It taps into live areas, including widening participation (WP) and diversifying the medical workforce to represent diverse UK populations/citizens..

It is timely as there is debate in HEIs around how to identify and support WP students without stigmatising them.

It could draw a bit more on issues for less privileged groups amongst medical students, such as the cost of living, working alongside studies (a compelling literature here).

I would value seeing more on what constitutes 'extracurricular' and how this could influence post FY opportunities- at the moment, this is perhaps a bit of a tenuous link for the reader to make. Could this be sharpened?

I also think that the association/correlation between extracurricular and awarding gap could be honed; it reads a bit as an after thought here?

There is also perhaps a lack of clarity between the fact that current UKFPO processes have removed the onus on extracurricular- this is skimmed over and we are led to post FY1/2.

When were these data collected? Apologies if I have missed a date. Was there a pandemic influence on extracurricular opportunities? Could this be a limitation?

What were the total number of medical students eligible to complete? The data set is large and impressive.

Female >2/3 of participants is skewed (we know females tend to respond more) and represents more females compared to national numbers of medical students (@60%).
The international participants is also greater than the 7.5% cap.

Prizes and merits are very complex and HEI dependent- I am not sure of the value of including this metric, it is not meaningful in my opinion- such a discrepancy between medical schools.

I was particularly interested that private school education led here to a greater number of research and leadership outputs. Is confidence a factor here?

The question of breakdowns of ethnic groups persists in all this sort of work- some groups are brand and diverse within the category and can be ratified further. Another potential limitation?

Most UK medical schools do offer 'spiral' learning and education around careers- this is not an original pedagogic idea, but reads as if it is.

I am not entirely sure that the 'so what' or this paper i.e. the potential application, lessons and impact are as clearly defined as they could be.

Finally, I would like to see something about how this sort of enquiry could lead to better support of students who may historically underrepresented in extracurricular activities (define better) for the benefit of workforce longevity and PATIENT CARE. Without extrapolating these finding to health of the workforce, equity and PATIENTS, I am not sure you are truly capitalising on the possible IMPACT of this interesting study.

Well done on an interesting piece of work.

**Reviewer**	**3**
**Name**	**Dack, Kyle**
**Affiliation**	**University of Bristol, MRC Integrative Epidemiology Unit**
**Date**	**16-Jan-2025**
**COI**	**None**

This manuscript explores the links between demographic characteristics and extracurricular activities, based on a survey of UK medical students.

There are significant flaws in the statistical techniques and reporting. The key issues are (a) not enough information in the methods concerning the survey, outcome, and statistical procedures (b) use of stepwise model selection which there is a strong consensus to avoid in epidemiology (c) lack of consideration for the risk of confounding bias and (d) a lack of context for why these characteristics were selected and what prior research tells us.

Given that addressing these would require a significant change to the analysis and full rewrite I recommend rejecting.

Abstract
1. “Secondary outcomes included analysing the influence of demographic factors — such as

gender, ethnicity, schooling type, and family background — on these achievements." I don't think "secondary outcomes" is the correct term here – because the outcome remains extracurricular activities – this sounds like a second analytical aim. It seems to me the outcome is extracurricular activities in medical students, which was then analysed by (a) identifying and describing and (b) evaluating against possible demographic predictors.

2. The results section is missing statistical detail which makes it harder to interpret - percentages, effect estimates, and confidence intervals or p-values where appropriate.

Introduction

3. Page 5 line 32. It would be helpful if the main results from references 7 or 8 could be reported, to allow the reader to notice any differences when reading the results.

4. P5 Line 40. "FAST study" is referenced but has not been explained yet – suggest adding a brief explanation including the acronym meaning.

5. P5 line 55. I can see that there is a second paper in review and a placeholder reference in both papers. But I cannot think of a way in which both papers can reference each other, one will be published before the other reference is known?

6. The second aim: "What are the implications of these disparities for specialty training applications and the broader medical profession?" does not appear in the abstract, and I'm not sure how the paper would answer it based on the methods and data reported in the abstract?

Methods

7. Page 7 line 8. FAST/AIM are missing explanation of the acronyms.

8. "study design" lines 13-26 are details of the survey design and measurement methods – this is more relevant to a subheading such as "outcome measurement" or "demographic characteristics" which I note in a later point are missing.

9. Participant recruitment: could the authors describe when/how the eligibility criteria were applied?

10. There is currently no sections describing the outcome or the demographic characteristics – how were they measured, how were they selected, what did they intend to represent, and what data transformations were applied. This is required for STROBE item 7/8.

11. STROBE items 9 is also not addressed as far as I can see? The checklist mentions pages 5/6 but I do not see any details of potential biases and how they were minimized.

12. Data analysis: There are a lot of details missing from this section. E.g. “Graphs and tables were generated using GraphPad Prism” – using which variables, to meet which aims? “To explore associations between demographic characteristics and students’ extracurricular activities” which characteristics? Up to this point there has been no comprehensive list of characteristics.

13. “Multivariable logistic regression was employed to assess the combined effect of multiple explanatory variables, selected using Akaike’s Information Criterion (AIC), to obtain the model that best balances complexity and predictive capability.” AIC is used typically to optimize the predictive power of a model towards future data, and it is not suitable for this type of inferential statistical approach. This approach does not minimize the risk of confounding, meaning there is a high risk that the results may be spurious and due to confounders or inappropriate mediators in the same model. Model selection should instead be based on existing theory and evidence, and what the authors consider to be the ideal model – which no model metric can determine. There is a variety of literature on covariate selection but I suggest this for an explanation of why AIC is not appropriate: https://www.stat.berkeley.edu/~aldous/157/Papers/shmueli.pdf

14. Goodness of fit is also mentioned but it is not explained how this statistic is used – again I recommend not using it at all, unless the model is intended as a future predictive tool.

15. P19 line 14-19. Please move to the discussion.

Results

16. Please make the number of decimal places consistent throughout the paper and results.

17. “There was a notably lower response rate from students in the ‘Year 4 (not penultimate year)’ category compared to other year groups” please report the numbers for this and any other findings throughout the results section.

18. Table 1. A major issue with the current manuscript is that this is the first time the demographic characteristics are described – but they should have been mentioned in the introduction, and in detail explained in the methods.

19. Extracurricular activities: similarly, no definition has been given for what this means or what is/isn’t considered a valid activity.

20. Table 2. What if somebody has done more than 1? Was this question multiple choice? If not, how were students asked to respond?

21. “Extracurricular achievements subanalysis” The statistical methodology is not explained – what model results are being reported? I am not sure why it is a called a subanalysis which may be part of the confusion. Please add a table of model results to the main paper – they are referenced extensively and it will be easier for the reader.

22. “Logistic regression analysis” – I thought the previous section may be logistic regression. If not, then the previous section was not explained in the methods.

23. “stepwise model selection based on Akaike’s information criterion (AIC) indicated that its inclusion did not significantly improve the model's predictive power”. Stepwise was not mentioned in the methods, but neither a stepwise approach nor using AIC are recommended because it fails to evaluate the risk of confounding.
https://ard.bmj.com/content/77/Suppl_2/5.1

24. The chi-square and degrees of freedom are not necessary to report, but please do add effect estimates (odds ratios) and confidence intervals.

Discussion
25. I do not think the results are likely to be valid given the statistical methods used, and this impacts much of the discussion.

VERSION 1 - AUTHOR RESPONSE

Reviewer 1

Comment 1: Include a sentence in the abstract outlining why extracurricular achievement is important, that is why it’s important to look at socioeconomic and demographic influences.

> Response: We have revised the abstract to include a sentence clarifying the relevance of extracurricular achievements in medical training and career progression, and the value of understanding socioeconomic and demographic influences for equity and widening participation.

Comment 2: While there is a recognition in the limitations section of the fact that attendance at private school is not necessarily indicative of socio-economic standing, this should be acknowledged earlier in the discussion.

> Response: Agreed. We have moved this acknowledgement to earlier in the discussion section to appropriately contextualise the limitations of using school type as a proxy for socioeconomic status.

Reviewer 2

Comment 1: It could draw a bit more on issues for less privileged groups amongst medical students, such as the cost of living, working alongside studies.

Response: We have expanded the discussion to include literature on the financial pressures faced by less privileged students, including the cost of living and the need to work alongside studies, and how these may impact participation in extracurricular activities.

Comment 2: I would value seeing more on what constitutes 'extracurricular' and how this could influence post-FY opportunities – at the moment, this is perhaps a bit of a tenuous link for the reader to make. Could this be sharpened?

Response: We have clarified what constitutes an extracurricular activity in our introduction and methods sections and strengthened the link between these achievements and post-foundation year opportunities in the introduction.

Comment 3: I also think that the association/correlation between extracurricular and awarding gap could be honed; it reads a bit as an afterthought here.

Response: We have revised the discussion to more clearly highlight the relationship between extracurricular participation and the awarding gap, framing this as an important equity consideration.

Comment 4: There is also perhaps a lack of clarity between the fact that current UKFPO processes have removed the onus on extracurricular – this is skimmed over and we are led to post-FY1/2.

Response: We have clarified the changes to UKFPO selection criteria and distinguished between the current and past relevance of extracurricular achievements, particularly regarding specialty training applications.

Comment 5: When were these data collected? Apologies if I have missed a date. Was there a pandemic influence on extracurricular opportunities? Could this be a limitation?

Response: The timeline can be found in the methods section. We believe the COVID pandemic had minimal, if any impact in EA, as there are no trends other than temporal.

Comment 6: What were the total number of medical students eligible to complete? The dataset is large and impressive.

Response: Due to the nature of survey dissemination, we are unable to determine a precise denominator or response rate. This has now been noted as a limitation.

Comment 7: Female >2/3 of participants is skewed (we know females tend to respond more) and represents more females compared to national numbers of medical students (~60%). The international participants are also greater than the 7.5% cap.

Response: We acknowledge the gender imbalance in our sample, with a higher proportion of female respondents than the national average. This reflects known trends in survey response rates, where female participants are typically overrepresented. Similarly, international students exceeded the 7.5% cap applied to UK medical school admissions, likely due to the inclusion of institutions that exclusively admit

international students (e.g., Brunel). We have added this to the limitations section and discussed how these factors may have influenced our findings and their generalisability.

Comment 8: Prizes and merits are very complex and HEI-dependent – I am not sure of the value of including this metric, as it is not meaningful in my opinion.

Response: We agree that the awarding of prizes and merits varies considerably between institutions. However, at the time of survey design and delivery, selection processes for multiple training programmes employed these as one of their selection criteria. The use of awards in selection processes has changed over time, but the present system seems to prefer those bestowed to only a small percentage for academic performance (such as the top 10-15% of students). However, other prizes may signal commitment to specialty: itself a separate criterion used in recruitment to some programmes such as ophthalmology. We now specify in the paper that all achievements about which we enquire were informed by published selection criteria.

Comment 9: I was particularly interested that private school education led to a greater number of research and leadership outputs. Is confidence a factor here?

Response: We have explored the potential role of confidence as a mediating factor in the relationship between private school education and research/leadership outputs.

Comment 10: The question of breakdowns of ethnic groups persists in all this sort of work – some groups are broad and diverse within the category and can be ratified further. Another potential limitation?

Response: While our analysis follows standard UK medical education categorisations to facilitate comparison with existing literature, we have acknowledged this as a limitation.

Comment 11: Most UK medical schools do offer 'spiral' learning and education around careers – this is not an original pedagogic idea but reads as if it is.

Response: We acknowledge that many medical schools offer some level of careers education and agree that this is not a new concept. However, we feel that career education delivered by UK medical schools in its current form is inadequate, so have revised the text to more accurately emphasise that existing career guidance needs to be significantly bolstered.

Comment 12: I am not entirely sure that the 'so what' of this paper – i.e., the potential application, lessons, and impact – are as clearly defined as they could be.

Response: We have revised the discussion to better articulate the implications of our findings, emphasising how they could inform policies to support underrepresented students, improve equity, and enhance workforce sustainability.

Comment 13: Finally, I would like to see something about how this sort of inquiry could lead to better support of students who may historically be underrepresented in extracurricular activities (define better) for the benefit of workforce longevity and patient care.

Response: We have included a section outlining strategies to better support underrepresented students, such as targeted mentoring and financial assistance, linking these to long-term benefits for workforce equity and patient care.

Reviewer 3:

Comment 1: There are significant flaws in the statistical techniques and reporting. The key issues are (a) not enough information in the methods concerning the survey, outcome, and statistical procedures (b) use of stepwise model selection which there is a strong consensus to avoid in epidemiology (c) lack of consideration for the risk of confounding bias and (d) a lack of context for why these characteristics were selected and what prior research tells us.

Response: We acknowledge these concerns and are committed to addressing them comprehensively:

a) We have expanded the methods section to include detailed descriptions of the survey design, outcome measurement, and statistical procedures. This ensures clarity and transparency, in full compliance with the STROBE checklist.
b) While stepwise model selection was employed to balance complexity and predictive capability, we recognise that its use is debated in the field. Although it is a common and widely used approach in observational studies, we have rerun the analysis using theory-driven covariate selection methods informed by prior research and established frameworks. This addresses concerns about robustness and ensure alignment with best practices in epidemiology.
c) We explicitly addressed potential confounding biases in the methods and limitations sections, identifying key factors that may influence both predictors and outcomes.
d) Additional context for the selection of demographic and socioeconomic characteristics has been provided, drawing on relevant literature to substantiate their inclusion and relevance to the research question.

Comment 2: Given that addressing these would require a significant change to the analysis and full rewrite I recommend rejecting.

Response: We appreciate the reviewer's concerns but respectfully note that the revised analysis did not require a full rewrite and was straightforward to implement, as it involved revisiting a single prediction model rather than altering the core results.

Comment 3. "Secondary outcomes included analysing the influence of demographic factors — such as gender, ethnicity, schooling type, and family background — on these achievements." I don't think "secondary outcomes" is the correct term here – because the outcome remains extracurricular activities – this sounds like a second analytical aim. It seems to me the outcome is extracurricular activities in medical students, which was then analysed by (a) identifying and describing and (b) evaluating against possible demographic predictors.

Response: Agreed. We have rephrased this throughout to clarify that extracurricular achievement is the sole outcome and demographic predictors are the focus of secondary analytical aims.

Comment 4. The results section is missing statistical detail which makes it harder to interpret - percentages, effect estimates, and confidence intervals or p-values where appropriate.

Response: This has been revised to include this information throughout.

Comment 5: Page 5 line 32. It would be helpful if the main results from references 7 or 8 could be reported, to allow the reader to notice any differences when reading the results.

Response: We have drawn more explicitly on the findings of these studies in the introduction to provide clearer context for our own results.

Comment 6. P5 Line 40. “FAST study” is referenced but has not been explained yet – suggest adding a brief explanation including the acronym meaning.

Response: These are now defined upon first use in the manuscript.

Comment 7. P5 line 55. I can see that there is a second paper in review and a placeholder reference in both papers. But I cannot think of a way in which both papers can reference each other, one will be published before the other reference is known?

Response: It is our hope that both papers are published in the same issue and therefore the reference can be pre-empted at the revision stage. We are of course prepared to remove the placeholder reference if this is not the case.

Comment 8. Page 7 line 8. FAST/AIM are missing explanation of the acronyms.

Response: These are now defined upon first use in the manuscript.

Comment 9. “study design” lines 13-26 are details of the survey design and measurement methods – this is more relevant to a subheading such as “outcome measurement” or “demographic characteristics” which I note in a later point are missing.

Response: The methods section has been rewritten to address all these points and comply fully with STROBE items 7–9.

Comment 10. Participant recruitment: could the authors describe when/how the eligibility criteria were applied?

Response: We have amended the methods section to explicitly state that all participants were required to confirm their active student status at a UK medical school – the key criteria for eligibility – in the first section of the questionnaire. The study timeline is also detailed in the methods, with reference made to the published study protocol for more information.

Comment 11. There are currently no sections describing the outcome or the demographic characteristics – how were they measured, how were they selected, what did they intend to represent, and what data transformations were applied? This is required for STROBE item 7/8.

Response: We have revised the methods section to include a clearer description of the outcome (extracurricular achievements) and the demographic characteristics. In view of the space constraints, we provide a concise summary of the survey design, implementation and rationale, and refer to our published protocol for a more detailed exposition.

Comment 12. STROBE item 9 is also not addressed as far as I can see? The checklist mentions pages 5/6 but I do not see any details of potential biases and how they were minimised.

> Response: We have rewritten the manuscript to comply with the STROBE guidelines. To avoid an overly formulaic style that disrupts the overall narrative, we have addressed these points in the limitations section.

Comment 13. Data analysis: There are a lot of details missing from this section. E.g. "Graphs and tables were generated using GraphPad Prism" – using which variables, to meet which aims? "To explore associations between demographic characteristics and students' extracurricular activities" which characteristics? Up to this point there has been no comprehensive list of characteristics.

> Response: We have rewritten the methods and data analysis sections to describe in detail the demographic information collected by the dataset (namely age, gender, schooling, fee status, ethnicity, family background and year of study), and the full list of extracurricular activities surveyed.

Comment 14. "Multivariable logistic regression was employed to assess the combined effect of multiple explanatory variables, selected using Akaike's Information Criterion (AIC), to obtain the model that best balances complexity and predictive capability." AIC is used typically to optimise the predictive power of a model towards future data, and it is not suitable for this type of inferential statistical approach. This approach does not minimise the risk of confounding, meaning there is a high risk that the results may be spurious and due to confounders or inappropriate mediators in the same model. Model selection should instead be based on existing theory and evidence, and what the authors consider to be the ideal model – which no model metric can determine. There is a variety of literature on covariate selection but I suggest this for an explanation of why AIC is not appropriate: https://www.stat.berkeley.edu/~aldous/157/Papers/shmueli.pdf

> Response: We acknowledge the limitations of stepwise model selection and AIC - failure to account for post-selection inference invalidates some confidence intervals and p-values in our previous approach. While data-driven variable selection is useful in other statistical contexts when performed judiciously, we now refrain from using it altogether, as the full dataset includes only a small number of demographic and educational predictors, whose inclusion we have justified carefully based on the existing literature. We also discuss potential unobserved confounders in the limitations section and avoid making causal conclusions in the absence of detailed information about the interplay between different socioeconomic factors. We interpret effect estimates and confidence intervals within the context of the working logistic model, which appears appropriate for our dataset based on scientific considerations and basic model diagnostics. This should alleviate concerns about a "high risk of spurious results due to confounders or inappropriate mediators." We emphasise that further research is needed to justify any causal statements, and that the results of any statistical analysis should be understood in light of the maxim, "all models are wrong, but some are useful."

Comment 15. Goodness of fit is also mentioned but it is not explained how this statistic is used – again I recommend not using it at all, unless the model is intended as a future predictive tool.

Response: Thank you for this suggestion — we no longer refer to goodness of fit in the paper.

Comment 16. P19 line 14-19. Please move to the discussion.

Response: We will move this content to the discussion section as recommended.

Comment 17. Please make the number of decimal places consistent throughout the paper and results.

Response: We have standardised the number of decimal places reported across all statistics to ensure consistency throughout the manuscript. As mentioned in the data analysis section, percentages are rounded to one decimal place. Odds ratios, confidence intervals and p-values are expressed to two decimal places, except those p-values below 0.01, which are either rounded to one significant figure or denoted as $p<0.0001$ where appropriate.

Comment 18. "There was a notably lower response rate from students in the 'Year 4 (not penultimate year)' category compared to other year groups" please report the numbers for this and any other findings throughout the results section.

Response: Response numbers are reported in full in Table 1. We have referenced previous works which showcase a similar pattern across intercalator groups/traditional course students, due to their lower numbers.

Comment 19. Table 1. A major issue with the current manuscript is that this is the first time the demographic characteristics are described – but they should have been mentioned in the introduction, and in detail explained in the methods.

Response: The study protocol, which we reference in the methods section, describes these in detail. However, we acknowledge the importance of making this clear within the manuscript itself, and have therefore updated the introduction and methods sections accordingly.

Comment 20. Extracurricular activities: similarly, no definition has been given for what this means or what is/isn't considered a valid activity.

Response: We have expanded upon our definition of extracurricular activities, specifying which activities are included and hope this is now clearer.

Comment 21. Table 2. What if somebody has done more than 1? Was this question multiple choice? If not, how were students asked to respond?

Response: Table 2 is not a 100% sum table. To ensure this is clear to the reader, we have explicitly stated in the text that respondents were free to select as many options as applied to them. We were keen to gather as much information as possible regarding their extracurricular achievements.

Comment 22. "Extracurricular achievements subanalysis" The statistical methodology is not explained – what model results are being reported? I am not sure why it is called a subanalysis

which may be part of the confusion. Please add a table of model results to the main paper – they are referenced extensively, and it will be easier for the reader.

> Response: Thank you for this comment and apologies for the lack of clarity and detail. We have rewritten the methods and data analysis sections to give a more concrete and transparent description of our statistical methodology. We originally used 'subanalysis' to refer to a breakdown of the student cohort by a particular demographic attribute such as gender or ethnicity but have now dropped this non-standard and potentially confusing terminology. While we understand the suggestion to include a table of model results in the main paper, we must balance the journal's table/figure limitations and the broader readership's needs. As such, we will retain the full model results in the supplementary material.

Comment 23. "Logistic regression analysis" – I thought the previous section may be logistic regression. If not, then the previous section was not explained in the methods.

> Response: We have expanded the methods and data analysis sections to describe the predictor and outcome variables in our logistic regression models, the full details of which are provided in the supplement. We agree that the single-variable odds ratios in our original manuscript were unsuitable due to confounding bias, so they have all been replaced with (adjusted) odds ratio estimates from multivariable logistic models containing all demographic and educational predictors.

Comment 24. "stepwise model selection based on Akaike's information criterion (AIC) indicated that its inclusion did not significantly improve the model's predictive power". Stepwise was not mentioned in the methods, but neither a stepwise approach nor using AIC are recommended because it fails to evaluate the risk of confounding. https://ard.bmj.com/content/77/Suppl_2/5.1

> Response: Thank you for raising the drawbacks of stepwise regression. Since there are only 8 categorical predictors in the full dataset, we now include them all in our multivariable logistic models and avoid data-driven variable selection altogether. It is true that our previous approach did not account for post-selection inference, and as such our the reported confidence intervals and p-values were not valid. These issues no longer arise, and we now mention explicitly in the data analysis section that a Bonferroni-adjusted significance threshold of $0.05/25 = 0.002$ is appropriate, since fewer than 25 hypothesis tests (multiple comparisons) are performed in the main text.

Comment 25. The chi-square and degrees of freedom are not necessary to report, but please do add effect estimates (odds ratios) and confidence intervals.

> Response: We have substantiated our findings in the results section with relevant effect estimates (i.e. odds ratios) and confidence intervals and removed unnecessary goodness-of-fit statistics.

Comment 26. I do not think the results are likely to be valid given the statistical methods used, and this impacts much of the discussion.

> Response: We respectfully disagree that the results are invalid. However, we recognise that the survey design and statistical methods could be refined to ensure the robustness of the findings and their causal interpretations. By adopting a scientifically informed

approach to covariate selection and addressing concerns about confounding, we settled on a simple and interpretable multivariable logistic model that includes all important covariates in the dataset. We have substantially revised the methods and analysis sections to reflect the improved methodology and provided full model output in the supplement for greater transparency.

VERSION 2 - REVIEW

**Reviewer**	**3**
**Name**	**Dack, Kyle**
**Affiliation**	**University of Bristol, MRC Integrative Epidemiology Unit**
**Date**	**15-Apr-2025**
**COI**	

Statistical review summary:

The authors have made a lot of improvements to the statistical analysis, and as a result I think there is much lower risk of bias from confounding or other sources.

There are a few minor suggestions below, the main one being to consider also presenting model results in table format.

Abstract:

1. "[…] those with a parent or sibling in medicine (OR 1.38, CI [1.12, 1.69], p=0.002) had notably higher participation in research" A small point but I suggest phrasing as "higher odds of participation" to make it clear to readers that this is the binary outcome.

Methods

2. Full dataset and missing values: I suggest this would be more suitable to the results? It is not really describing any methods used by the study.

3. Data analysis: this is much clearer and I think the new approach will produce more generalizable results. One thing which is currently missing is related to STROBE item 9 – efforts to minimize bias. In particular confounding bias is not mentioned. It sounds like the authors have used a predictor set which will reduce the risk of bias from confounding factors, but I recommend to directly state this – because this is very often an item on quality assessment tools and could impact how the study is evaluated in any future reviews or evidence summaries.

Results

4. Table 2. The authors clarified that this does not sum to 100%, and this was also clarified in the methods. I suggest reminding readers of this when it is introduced in the results text,

e.g. (multiple options possible) or similar, because at a glance it does look like it sums to 100%, even though it is much higher. I misinterpreted this table so other readers may also.

5. Tables for the multivariable regression results would be helpful. These are the headline results reported in the abstract and discussion, so having a table would make it easier for readers to see – even if in the supplementary materials. Two figures are provided but this does not provide the same detail as a results table. Examples can be found here in tables 3 and 4: https://bmjopen.bmj.com/content/9/9/e028555.long

---

VERSION 2 - AUTHOR RESPONSE

Reviewer: Dr Kyle Dack
Comment 1:
"A small point but I suggest phrasing as 'higher odds of participation' to make it clear to readers that this is the binary outcome."
Response:
This sentence has been amended. Thank you.

Comment 2:
"Full dataset and missing values: I suggest this would be more suitable to the results? It is not really describing any methods used by the study."
Response: We agree and have relocated this sentence to the Results section.

Comment 3:
"Efforts to minimise bias (STROBE item 9)… it sounds like the authors have used a predictor set which will reduce the risk of bias from confounding factors, but I recommend to directly state this."
Response:
We have added the following to the end of the Data Analysis section:
"All predictor variables were included simultaneously in each model, using appropriate baseline categories to minimise the risk of confounding bias and strengthen the internal validity of our findings."

Comment 4:
"Table 2: I suggest reminding readers that multiple options were possible, because it initially appears to sum to 100%."
Response: We have clarified this in the Results text when Table 2 is first introduced. It now reads:
"Percentages in Table 2 reflect the proportion of respondents reporting participation in each activity. As multiple selections were permitted, totals exceed 100%."

Comment 5:
"Tables for the multivariable regression results would be helpful… even if in the supplementary materials."
Response: We have added a new Table 3 to the main manuscript, presenting multivariable regression results for (a) overall extracurricular participation and (b) research authorship side by side. This mirrors the style of article provided as suggested.
